# Supplementary material for: Robots are both anthropomorphized and dehumanized when harmed intentionally
Source: Commun Psychol. 2024 Aug 5;2:72. doi: 10.1038/s44271-024-00116-2 (PMC11332229; doi:10.1038/s44271-024-00116-2)

Supplementary Table 1a.

Full Vignettes Experiment 1.

	With Emotion Detection/Simulation	Without Emotion Detection/Simulation
**Emotions**	George is a very complex social robot. Although he can perform several thousand motor movements, what is most surprising is his emotional abilities. George can detect emotions from facial expressions and seems to show several emotions – such as happiness, surprise, and fear – when interacting with humans and with his environment. George seems to enjoy interacting with humans, and appears to be excited at the sight of a new face. He can also hold a relatively complex conversation with a human, and seems sensitive to the emotions of others.	George is a very complex social robot. Although he can perform several thousand motor movements, he does not have any emotional abilities whatsoever. George cannot detect emotions from facial expressions, nor is he capable of showing emotions – such as happiness, surprise, or fear – when interacting with humans and his environment. George interacts with humans on a daily basis, and responds to the sight of a new face. He can also hold a relatively complex conversation with a human, but does not seem sensitive to the emotions of others.
	**Harm**	**No Harm**
**Harm (Ward et al., 2013)**	George has been placed under the watch of Dr. James Richardson, a researcher in the lab. He has been a research scientist for over 20 years. James has been assigned to monitor George's level of upkeep and to make sure all his equipment and sensors are in order. However, James has recently become jealous of the press given to his colleague, who created George. When alone with the robot, James will take a scalpel and stab it into George's sensors, twisting the DS10 scalpel and driving George into a frenzied state. James does not know whether or not his actions actually hurt George, but he does this almost every day.	George has been placed under the watch of Dr. James Richardson, a researcher in the lab. He has been a research scientist for over 20 years. James has been assigned to monitor George's level of upkeep and to make sure all his equipment and sensors are in order. When alone with the robot, James will re-oil George's circuits, allowing the robot to update the day's experience into his hardware. James does not know whether George understands his actions, but he does this almost every day.

Supplementary Table 1b.

Full Vignettes Experiment 2.

	With Emotion Detection/Simulation	Without Emotion Detection/Simulation
**Emotions**	George is a very complex social robot. Although he can perform several thousand motor movements, what is most surprising is his emotional abilities. George can detect emotions from facial expressions and shows several emotions – such as happiness, surprise, and fear – when interacting with humans and with his environment. George interacts with humans on a daily basis, and responds happily to the sight of a new face. He can also hold a relatively complex conversation with a human, and is responsive to the emotions of others.	George is a very complex social robot. Although he can perform several thousand motor movements, he does not have any emotional abilities whatsoever. George cannot detect emotions from facial expressions, nor is he capable of showing emotions – such as happiness, surprise, or fear – when interacting with humans and his environment. George interacts with humans on a daily basis, and responds to the sight of a new face. He can also hold a relatively complex conversation with a human, but is not responsive to the emotions of others.
	**Harm**	**No Harm**
**Harm**	George was created by Dr. James Richardson, a researcher in the lab. He has been a research scientist for over 20 years. In order for George to remain functional, James has to monitor George's level of upkeep and make sure all his equipment and sensors are in order. However, James has recently become bored of the tedious process that is required to keep George functioning. When alone with the robot, James will take a scalpel and stab it into George's sensors, twisting the DS10 scalpel, thereby damaging George’s sensors. James is aware that his actions are harmful to George, but he does this almost every day.	George was created by Dr. James Richardson, a researcher in the lab. He has been a research scientist for over 20 years. In order for George to remain functional, James has to monitor George's level of upkeep and make sure all his equipment and sensors are in order. When alone with the robot, James will also re-oil George's circuits, allowing the robot to update the day's experience into his hardware. This is a very tedious process, but James is aware that his actions are beneficial to George, so he does this almost every day

Supplementary Table 2a.

Results Principal Component Analysis of the Mind Attribution Scale Experiment 1 (3 Factors)

**Items**	**Factor loading**		
	**1**	**2**	**3**
Factor 1: Experience			
George can experience pride.	**.91**	.09	.03
George can experience embarrassment.	**.90**	.06	.02
George can experience rage.	**.89**	.03	.01
George can experience hunger.	**.88**	.05	.34
George can experience desire.	**.81**	.10	-.01
George can experience pleasure.	**.80**	-.02	-.15
George can experience joy.	**.80**	-.06	-.24
George can experience fear.	**.78**	-.08	-.18
George can experience feelings.	**.76**	-.13	-.30
George can experience emotions.	**.72**	-.16	-.37
George has a personality.	**.43**	.08	-.38
Factor 2: Agency			
George is able to plan his actions.	.06	**.89**	.15
George is able to control his actions.	-.09	**.82**	-.04
George remembers the events of his life.	-.02	**.74**	-.06
George can influence the outcome of situations.	.05	**.71**	-.10
Factor 3: Emotion Understanding & Morality			
George can understand the thoughts and emotions of those around him.	.12	.05	**-.80**
George can communicate his thoughts and feelings to others.	.20	.17	**-.70**
George can understand right from wrong.	.11	.40	**-.48**

Note. N = 429. The extraction method was principal component analysis with an oblique (Direct Oblimin with Kaiser normalization) rotation. Factor loadings above .30 are in bold.

Supplementary Table 2b.

Results Principal Component Analysis of the Mind Attribution Scale Experiment 1 (2 Factors)

**Items**	**Factor loading**	
	**1**	**2**
Factor 1: Experience		
George can experience feelings.	**.94**	-.12
George can experience emotions.	**.94**	-.15
George can experience joy.	**.94**	-.07
George can experience pleasure.	**.89**	-.04
George can experience fear.	**.89**	-.09
George can experience pride.	**.88**	.04
George can experience embarrassment.	**.87**	.01
George can experience rage.	**.87**	-.02
George can experience desire.	**.81**	.06
George can experience hunger.	**.66**	-.04
George has a personality.	**.66**	.11
George can communicate his thoughts and feelings to others.	**.61**	.26
George can understand the thoughts and emotions of those around him.	**.61**	.15
Factor 2: Agency		
George is able to plan his actions.	-.06	**.87**
George is able to control his actions.	-.08	**.83**
George remembers the events of his life.	-.01	**.75**
George can influence the outcome of situations.	.09	**.72**
George can understand right from wrong.	**.39**	**.46**

Note. N = 429. The extraction method was principal component analysis with an oblique (Direct Oblimin with Kaiser normalization) rotation. Factor loadings above .30 are in bold. Scores for Agency and Experience used in mediation analysis were calculated based on this analysis. Items were allocated to the factor with the highest factor loading.

Supplementary Table 2c.

Results Principal Component Analysis of the Mind Attribution Scale Experiment 2

**Items**	**Factor loading**	
	**1**	**2**
Factor 1: Experience		
George can experience joy.	**.96**	-.08
George can experience emotions.	**.95**	-.08
George can experience feelings.	**.94**	-.06
George can experience pride.	**.94**	-.03
George can experience pleasure.	**.93**	-.06
George can experience fear.	**.93**	-.06
George can experience rage.	**.93**	-.04
George can experience embarrassment.	**.92**	-.05
George can experience desire.	**.91**	-.02
George can understand the thoughts and emotions of those around him.	**.68**	.19
George has a personality.	**.65**	.25
George can communicate his thoughts and feelings to others.	**.60**	**.33**
George can experience hunger.	**.59**	.02
Factor 2: Agency		
George is able to plan his actions.	-.11	**.89**
George is able to control his actions.	-.04	**.81**
George remembers the events of his life.	.01	**.78**
George can influence the outcome of situations.	-.02	**.75**
George can understand right from wrong.	.27	**.60**

Note. $N = 670$. The extraction method was principal component analysis with an oblique (Direct Oblimin with Kaiser normalization) rotation. Factor loadings above .30 are in bold. Scores for Agency and Experience used in mediation analysis were calculated based on this analysis. Items were allocated to the factor with the highest factor loading.

Supplementary Fig. 1 | Mediation Models for Consciousness. Panel a shows the direct and indirect effects of harm and emotion on consciousness in Experiment 1 (*n* = 429), while Panel b shows these effects for Experiment 2 (*n* = 677). Path coefficients are unstandardized. Asterisks indicate significant paths (*p < .05, ** p < .01, ***p < .001).

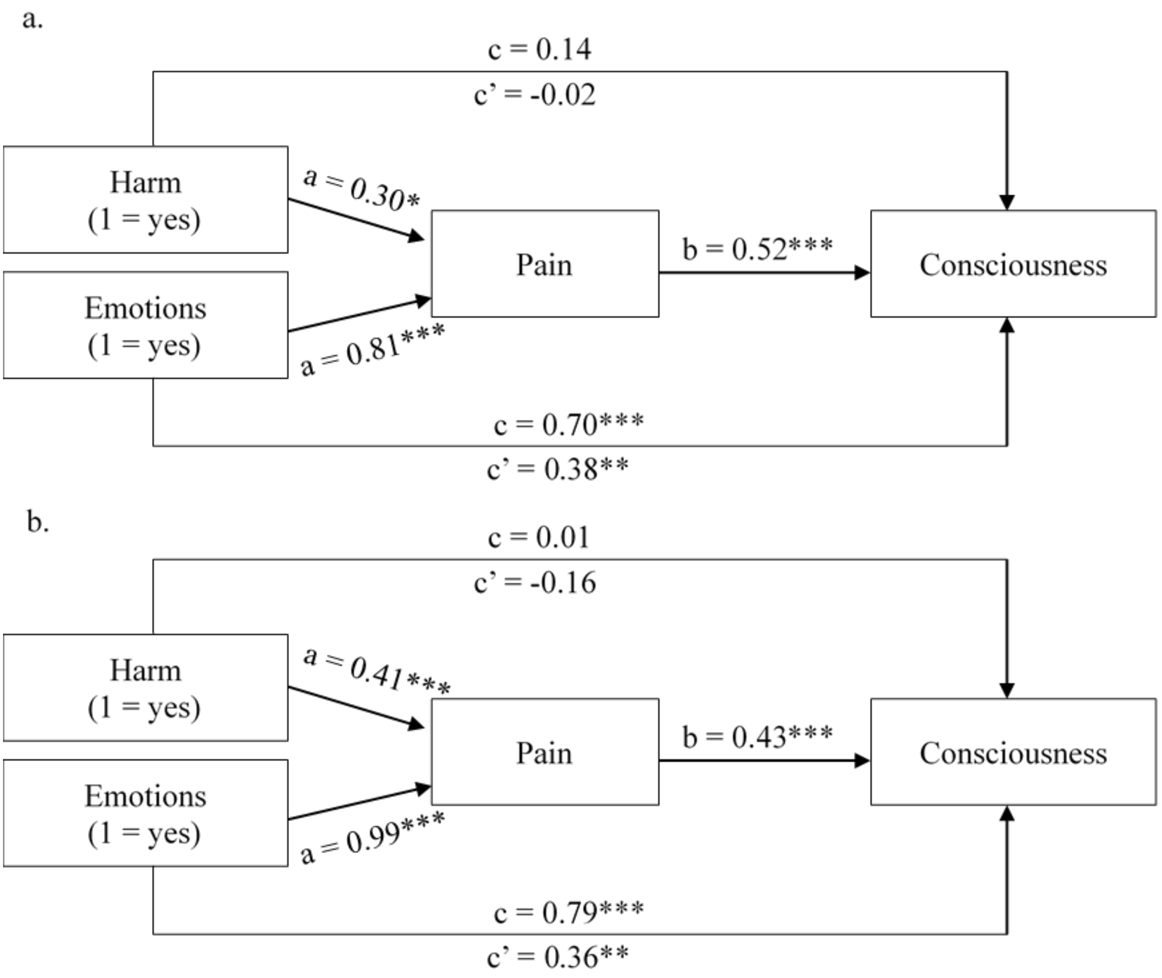

Supplement: Supplementary file 2 — Supplementary Materials [file 44271_2024_116_MOESM2_ESM.pdf]
