# Supplementary material for: Robots are both anthropomorphized and dehumanized when harmed intentionally
Source: Commun Psychol. 2024 Aug 5;2:72. doi: 10.1038/s44271-024-00116-2 (PMC11332229; doi:10.1038/s44271-024-00116-2)
Supplement: Supplementary file 3 — Reporting summary [file 44271_2024_116_MOESM3_ESM.pdf]

nature portfolio

Corresponding author(s): Marieke S. Wieringa

Last updated by author(s): 14-06-2024

Reporting Summary

Nature Portfolio wishes to improve the reproducibility of the work that we publish. This form provides structure for consistency and transparency in reporting. For further information on Nature Portfolio policies, see our Editorial Policies and the Editorial Policy Checklist.

Statistics

For all statistical analyses, confirm that the following items are present in the figure legend, table legend, main text, or Methods section.

n/a	Confirmed	
☐	☒	The exact sample size (*n*) for each experimental group/condition, given as a discrete number and unit of measurement
☐	☒	A statement on whether measurements were taken from distinct samples or whether the same sample was measured repeatedly
☐	☒	The statistical test(s) used AND whether they are one- or two-sided<br>*Only common tests should be described solely by name; describe more complex techniques in the Methods section.*
☒	☐	A description of all covariates tested
☐	☒	A description of any assumptions or corrections, such as tests of normality and adjustment for multiple comparisons
☐	☒	A full description of the statistical parameters including central tendency (e.g. means) or other basic estimates (e.g. regression coefficient) AND variation (e.g. standard deviation) or associated estimates of uncertainty (e.g. confidence intervals)
☐	☒	For null hypothesis testing, the test statistic (e.g. *F*, *t*, *r*) with confidence intervals, effect sizes, degrees of freedom and *P* value noted<br>*Give P values as exact values whenever suitable.*
☐	☒	For Bayesian analysis, information on the choice of priors and Markov chain Monte Carlo settings
☒	☐	For hierarchical and complex designs, identification of the appropriate level for tests and full reporting of outcomes
☐	☒	Estimates of effect sizes (e.g. Cohen's *d*, Pearson's *r*), indicating how they were calculated

Our web collection on statistics for biologists contains articles on many of the points above.

Software and code

Policy information about availability of computer code

Data collection	No software was used
Data analysis	We used statistical software SPSS (version 27) to analyse the data.

For manuscripts utilizing custom algorithms or software that are central to the research but not yet described in published literature, software must be made available to editors and reviewers. We strongly encourage code deposition in a community repository (e.g. GitHub). See the Nature Portfolio guidelines for submitting code & software for further information.

Data

Policy information about availability of data

All manuscripts must include a data availability statement. This statement should provide the following information, where applicable:

- Accession codes, unique identifiers, or web links for publicly available datasets
- A description of any restrictions on data availability
- For clinical datasets or third party data, please ensure that the statement adheres to our policy

All data associated with this project is published on the OSF platform.

Human research participants

Policy information about studies involving human research participants and Sex and Gender in Research.

Reporting on sex and gender	We only asked participants to indicate their gender, using answer categories "Male", "Female" and "Other". We did not perform any gender-based analyses, as the harm-made mind effect is not known to be affected by gender.
Population characteristics	See below.
Recruitment	Participants were recruited through the online recruitment platform Prolific.
Ethics oversight	Ethics Committee Social Sciences (ECSS) of Radboud University

Note that full information on the approval of the study protocol must also be provided in the manuscript.

Field-specific reporting

Please select the one below that is the best fit for your research. If you are not sure, read the appropriate sections before making your selection.

☐ Life sciences ☒ Behavioural & social sciences ☐ Ecological, evolutionary & environmental sciences

For a reference copy of the document with all sections, see nature.com/documents/nr-reporting-summary-flat.pdf

Behavioural & social sciences study design

All studies must disclose on these points even when the disclosure is negative.

Study description	The paper presents two experimental studies, both using a 2x2 between-subjects design.
Research sample	Participants were recruited using Prolific. Study 1 was available on all available countries in Prolific, fluent English was set as a pre-screening criterium. The final sample consisted of 429 participants (51% male, 48% female, 1% other, mean age = 28 years, SD = 9.33, range 18-66 years). Study 2 was also open to all available countries in Prolific, but we set English as a first language as a pre-screening criterium, as well as the exclusion of participants from Study 1. The final sample included 677 participants (38% male, 61% female, 1% other, mean age = 34.59, SD = 12.27, range 18-75 years). We have no further information on nationality or ethnicity of the participants.
Sampling strategy	The minimum sample size for study 1 was based on a calculation in G*power for replicating the smallest reported effect size in Ward et al. (2013), d = 0.38 (which corresponds to f = 0.19 for an ANOVA), using an alpha level of .95 and 90% power. The sample size for study 2 was based on a calculation in G*power using the effect sizes of Study 1 and a minimum of 80% power.
Data collection	Data were collected via an online survey programmed in Qualtrics.
Timing	Data for Study 1 were collected in August 2020. Data for Study 2 were collected in October 2020.
Data exclusions	For Study 1, we excluded 22 participants because they filled out the questionnaire in less than half of the median response time (i.e., less than 134.25 seconds). Another participant was excluded because their session failed to time-out after the maximum allowed response time of 30 minutes was exceeded. For Study 2, 21 participants were excluded because they filled out the questionnaire in less than half of the median response time, as described in the preregistration for this experiment. Additionally, another two participants were dropped because they indicated that they were younger than 18 years of age.
Non-participation	No participantes dropped out/declined consent to participate.
Randomization	To randomize the participants, we used the randomizer in Qualtrics and enabled the option to evenly distribute participants between groups.

Reporting for specific materials, systems and methods

We require information from authors about some types of materials, experimental systems and methods used in many studies. Here, indicate whether each material, system or method listed is relevant to your study. If you are not sure if a list item applies to your research, read the appropriate section before selecting a response.

Materials & experimental systems

n/a	Involved in the study
☒	☐ Antibodies
☒	☐ Eukaryotic cell lines
☒	☐ Palaeontology and archaeology
☒	☐ Animals and other organisms
☒	☐ Clinical data
☒	☐ Dual use research of concern

Methods

n/a	Involved in the study
☒	☐ ChIP-seq
☒	☐ Flow cytometry
☒	☐ MRI-based neuroimaging
